# Discovery of eQTL Alleles Associated with Autism Spectrum Disorder: A Case–Control Study

**DOI:** 10.1007/s10803-022-05631-x

**Published:** 2022-06-23

**Authors:** Allison R. Hickman, Bradley Selee, Rini Pauly, Benafsh Husain, Yuqing Hang, Frank Alex Feltus

**Affiliations:** 1 Genetics and Biochemistry Department, Clemson University, Clemson, SC 29634 USA; 2 Electrical and Computer Engineering Department, Clemson University, Clemson, SC 29634 USA; 3 Biomedical Data Science & Informatics Program, Clemson University, Clemson, SC 29634 USA; 4 Center for Human Genetics, Clemson University, Greenwood, SC 29646 USA; 5 Biosystems Research Complex, 302C, 105 Collings St, Clemson, SC 29634 USA

**Keywords:** Autism spectrum disorder, eQTLs, Classification, Neural network

## Abstract

Autism Spectrum Disorder (ASD) is a complex neurodevelopmental disorder characterized by challenges in social communication as well as repetitive or restrictive behaviors. Many genetic associations with ASD have been identified, but most associations occur in a fraction of the ASD population. Here, we searched for eQTL-associated DNA variants with significantly different allele distributions between ASD-affected and control. Thirty significant DNA variants associated with 174 tissue-specific eQTLs from ASD individuals in the SPARK project were identified. Several significant variants fell within brain-specific regulatory regions or had been associated with a significant change in gene expression in the brain. These eQTLs are a new class of biomarkers that could control the myriad of brain and non-brain phenotypic traits seen in ASD-affected individuals.

**Supplementary Information:**

The online version contains supplementary material available at 10.1007/s10803-022-05631-x.

## Introduction

Autism Spectrum Disorder (ASD) is a neurodevelopmental disorder caused by a combination of genetic and environmental factors resulting in a range of various phenotypes across ASD-affected individuals (Lai et al., 2014). The US Center for Disease Control estimates that 1 in every 44 people have ASD with more males affected than females at a 4:1 ratio (Maenner et al., 2021). While ASD can be diagnosed as early as 2 years of age, diagnosis usually occurs around the age of four (Baio, 2018). Subtraits, including repetitive behavior, challenges in social settings, and sensory issues will vary among ASD-affected individuals in their presentation and in severity (Lai et al., 2014). Comorbidities that occur with ASD can include brain-based conditions, such as epilepsy or anxiety, as well as non-brain-based conditions, such as inflammatory bowel disease and cardiac dysrhythmia (Doshi-Velez et al., 2014; Lord et al., 2020; Somekh et al., 2016). Environmental risks can include increased paternal age, birth trauma, and caesarean section delivery (Modabbernia et al., 2017; Wu et al., 2017a; Wu et al., 2017b). In addition to environmental risks, many molecular variations have been associated with ASD.

Complex human phenotypes involve allelic variation across multiple genes (Geschwind, 2008; Tang & Siegmund, 2002). ASD is clearly a complex trait where as many as 1,000 genes have been associated with the phenotype as found in the Simons Foundation Autism Research Initiative (SFARI) Gene database (Abrahams et al., 2013). Genomic features ranging in size from large DNA copy number variants (CNV) to single nucleotide variants (SNV) have been associated with the disorder (Geschwind, 2011; Lai et al., 2014; State & Levitt, 2011). CNVs, large-scale duplications or deletions, have an impact on gene dosage and can result in haploinsufficiency or altered transcription patterns (Sebat et al., 2007). Present in up to 10% of ASD-affected individuals, CNVs have been associated with genes SHANK2, SYNGAP1, DLGAP2, and others (Pinto et al., 2010). These can also occur in large chromosomal regions, such as 15q11-13, 16p11.2, and 22q11.2 (Geschwind, 2011; Velinov, 2019). Single gene mutations have also been identified, some of which as a result of comorbidity, such as FMR1 for fragile X syndrome and MECP2 for Rett syndrome (Betancur, 2011). For idiopathic cases, mutations have been identified in genes associated with synaptogenesis, like neuroligins (NLGN3, NLGN4), shank proteins (SHANK2, SHANK3), and neurexins (CNTNAP2, NRXN1) (Berkel et al., 2010; Durand et al., 2007; Jamain et al., 2003; Tan et al., 2010; Tromp et al., 2021). However, among these associations and others, no single association accounts for more than 2% of all cases (Abrahams & Geschwind, 2008). It is possible that for most ASD-affected individuals, the cause is more likely to be a network of altered genetic interactions rather than singular change with a global effect.

In addition to allelic variation in gene dosage and protein coding regions, it is likely that many human traits are modulated by variation in expression of genes within gene regulatory networks (GRNs) (Boyle et al., 2017). One technique to detect variation in gene output is through expression quantitative trait locus (eQTL) analysis by which a DNA variant at a specific genomic position is associated with the RNA expression level of a gene in a tissue-specific context (Dimas et al., 2009). eQTLs are identified through genome sequencing and transcriptome analysis, via microarray or RNA-seq, where RNA expression levels of each gene are tested for association with DNA polymorphisms across individuals in a defined DNA window surrounding the locus (Albert & Kruglyak, 2015). eQTLs afford a better understanding of natural variation and disease by offering a mechanistic explanation for variant alleles that segregate with a phenotype. With the publication of normal human tissue-specific eQTLs, such as those described by the Genotype-Tissue Expression (GTEx) project (GTEx Consortium, 2015), one can generate tissue-specific gene expression phenotype control hypotheses using genotype calls in affected and unaffected groups without the need of destructive sampling of human tissue.

eQTLs have previously been employed in ASD research. Sun *et al.* combined ASD GWAS SNV genotypes with frontal cortex RNA gene expression to identify eQTLs with altered expression levels across 76 genes between ASD-affected and control individuals (Sun et al., 2019). Most eQTLs were found to influence regulation due to their overlap with histone marks or an association with the methylation level of each associated gene. Other studies have uncovered multiple variants associated with expression levels of a particular gene. LoParo and Waldman looked exclusively at the oxytocin receptor gene (OXTR) and found four SNVs (three intronic and one in the promoter) in which an allele was associated with a higher risk of ASD (LoParo & Waldman, 2015). Wu *et al.* found three significant SNVs associated with ASD in the transcription start site of the MEGF10 gene, which is thought to be active in modulating neural connections during development (Wu et al., 2017a; 2017b).

By expanding polymorphism searches into intergenic genomic regions through whole genome sequencing, as opposed to exome sequencing, there are likely to be many genotype–phenotype associations to uncover that function in gene expression control. However, because of this widened scope, new techniques including machine learning may be necessary for the detection of genetic associations with ASD. Artificial neural networks (ANNs) are a machine learning technique used to detect patterns within an input of features (e.g. histology images, gene expression profiles, clinical data) and are capable of classifying labeled groups (Hyde et al., 2019). Deep learning ANNs, especially the Multi-Layer Perceptron (MLP) model, have been shown to be more effective in predicting disease compared to other traditional methods such as logistic regression models (Yu et al., 2019). MLPs were first used in 2001 to distinguish an individual’s specific type of small, round blue cell tumor based on their gene expression profile (Khan et al., 2001). Because the initial application of ANNs was in image analysis (Egmont-Petersen et al., 2002), it is not surprising that a large majority of ASD-related machine learning studies have been completed on brain imaging data (Bi et al., 2018; Sherkatghanad et al., 2020). A valuable resource used in many of these studies is the ASD brain imaging data exchange database, which contains structural and functional imaging data from roughly 2,000 individuals, half of which are ASD-affected (Di Martino et al., 2017). The projects employing this data tend to have classification accuracies ranging from 70 to 90% (Bi et al., 2018).

There are fewer studies that have used DNA sequence patterns as input to machine learning methods. Jiao *et al.* used the genotypes of 29 SNVs to predict symptom severity (mild, moderate, severe) among individuals diagnosed with ASD (Jiao et al., 2012). The SNVs fell within the introns, exons, promoters, or untranslated regions of nine genes that had previously been associated with ASD subtraits. This model was able to accurately predict the symptom severity in 67% of individuals (Jiao et al., 2012). Another study used DNA sequencing data as input into an ANN to measure the impact of a variant on splicing patterns between individuals with and without ASD (Xiong et al., 2015). Although the number of variants between the two groups did not significantly differ, the variants within the ASD-affected individuals were associated with more brain-expressed genes compared to controls, as well as a higher enrichment for brain development and function (Xiong et al., 2015).

A geometric driver of genetic discovery for ASD and other phenotypes are databases filled with huge numbers of genotyped individuals who are often tagged with rich phenotypic data. The Simons Foundation Powering Autism Research (SPARK) dataset published by SFARI contains DNA sequencing and phenotypic information for 27,615 individuals, including 9,779 affected individuals and their immediate family members (Feliciano et al., 2018). The sequencing data for each individual includes exome sequencing and whole genome genotyping performed on a saliva sample, while the phenotypic data includes an individual’s background history, a basic medical screening, a developmental coordination disorder questionnaire, a repetitive behavior questionnaire, and a social communication questionnaire (Feliciano et al., 2018). This dataset has allowed for new insights and discoveries in the field of ASD research (Bhat, 2021; Chang & Kochel, 2020; Matoba et al., 2020).

Additional databases contain genotyped individuals that serve to estimate allele frequencies and can be used to control for confounding population structure based on known ancestry. The Genome Aggregation Database (gnomAD) is a resource of genetic information spanning 105 million indels and 602 million SNVs produced by whole genome sequencing of 71,702 individuals (Karczewski et al., 2020). Over 52 projects have contributed to this dataset including the 1000 Genomes Project (Auton et al., 2015) and GTEx (GTEx Consortium, 2015). While gnomAD has published genotype summary information for many populations, GTEx has completed whole genome sequencing for a total of 818 Caucasian and African American individuals (phs000424.v8.p2) and contains individual-specific genotyping information available for analysis. Together, the gnomAD and GTEx datasets contain both population-specific and individual-specific genotype data to compare with populations of interest, like those of ASD-affected individuals (An et al., 2020; Novelli et al., 2020).

Another goal for GTEx was to publish tissue-specific gene expression data in relation to *cis*-regulation variants. In the eighth version of the database, there are 17,382 tissue samples, 15,201 of which underwent eQTL analysis. While GTEx investigated tissues across major human organs, PsychENCODE is a genomics database that focuses solely on brain features (Wang et al., 2018). These derived brain-specific lists include enhancers specific to the pre-frontal cortex, acetylation peaks specific to the pre-frontal cortex, temporal cortex, and cerebellar cortex, active brain eQTLs, and differential gene expression results comparing ASD-affected individuals to controls (Wang et al., 2018). These gene regulation data available through GTEx and PsychENCODE have been used to further investigate ASD (Chau et al., 2021; Pain et al., 2019; Reilly et al., 2020). Chau et al. used the BrainSpan RNA-seq dataset published by PsychENCODE to find that loss-of-function mutations were present in isoforms and were expressed at higher levels in ASD-affected individuals prenatally compared to controls (Chau et al., 2021; Wang et al., 2018). Others used information from GTEx, along with other datasets, to identify 14 differentially expressed genes in ASD and to find common trends in pathways and functions among ASD genes (Pain et al., 2019; Reilly et al., 2020).

Many large effect genetic associations have been identified in ASD-affected individuals, such as the genes SHANK3 and MECP2 as well as the CNV regions 15q11.2 and 16p11.2 (Abrahams et al., 2013). However, these large effects can only be identified in roughly 20% of the ASD population (Lord et al., 2020). It is possible that for most ASD-affected individuals, the disorder stems from the accumulation of many small effect changes (Klei et al., 2012). eQTLs are one small effect genetic feature that has not been fully investigated on a genome-wide scale in relation to ASD. In this study, we used tissue-specific eQTLs as our search space for genetic associations with ASD. While some studies have investigated eQTLs surrounding specific genes, we have not limited our search to genes already associated with the brain or with ASD (LoParo & Waldman, 2015; Wu et al., 2017a; 2017b). After discovering eQTL alleles associated with ASD, we employ machine learning models to test their classification accuracy.

## Methods

### GTEx eQTLs

 Tissue-specific *cis*-eQTLs (version 8) were downloaded from the GTEx website [https://gtexportal.org/home/datasets]. The most significantly associated variant for each gene in each tissue was considered for analysis. The GTEx eQTLs with a qval < 0.05 were retained, as recommended by GTEx. From these eQTLs, we were first interested in the associated variants. The variant information included the locus (chromosome and base position), as well as the reference and alternate alleles. Here, each variant was listed as ‘chromosome_base-pair_reference-allele_alternate-allele’. Of the eQTLs meeting the qval criteria, a list of variants was extracted.

### Genetic Dataset Overview

This study used genetic data from the Simons Foundation Autism Research Initiative SPARK Regeneron dataset released May 1, 2019, which included whole-exome sequencing data, whole-genome genotyping data, background information, and surveys for the ASD-affected individuals and their families. For the two outside control groups, individual-level genotypes and background information were obtained from GTEx (phs000424.v8.p2) and population-level genotypes were accessed from gnomAD (v3.1). To protect this data, the authors were granted access to the GTEx and SPARK datasets through IRB approval and everyone on the project underwent human subjects training. The data was stored on a private allocation on Clemson’s Palmetto Cluster with access only available to those directly working on it. All analyses were also completed on that allocation to protect the data from other users.

### SPARK Dataset

Because of the genetic nature of the study, ASD-affected individuals with possible environmental contributions to their diagnosis were excluded. These conditions included: birth or pregnancy complications, premature birth, fetal alcohol syndrome, and cognitive delays due to exposure or medical condition. Due to genetic similarities in families, only one individual from each family was included in this study. We limited this study to ASD-affected individuals with two ASD-unaffected parents, from Caucasian or African American populations, excluding those with mixed race. After taking these limitations into account there were 1,647 Caucasian ASD-affected individuals (1,300 males, 347 females) and 48 ASD-affected African American individuals (40 males, 8 females) available for study.

### GTEx and gnomAD Datasets

The GTEx VCF data included genotype calls for a total of 838 people, including 715 Caucasian (472 males, 243 females) and 103 African American individuals (71 males, 32 females). For the gnomAD dataset, the ‘Non-Neuro-NFE’ subset was used, which included non-Finnish European individuals who were either not enrolled in a neurological study or were designated as a control in a neurological study (N = 37,543).

### Filtering VCFs

For SPARK and GTEx VCFs, genotype calls for each individual were filtered for depth and genotype quality (DP > 10, GQ > 20) using bcftools. The genotype calls that did not meet both thresholds were considered a no-call. VCFs were then transformed into genotype matrices using JVARKIT tools published by Pierre Lindenbaum (https://github.com/lindenb/jvarkit), where each variant was represented as a ‘0’ for a homozygous reference allele genotype, a ‘1’ for a heterozygous genotype, a ‘2’ for a homozygous alternate allele genotype, and a ‘−1’ for a no-call. From these matrices the individuals within the Discovery and Classification subsets and their genotypes at eQTL loci were extracted. Each variant within these subsets had to have genotype calls for at least 90% of the individuals of interest in each Discovery and Classification subset to be further considered.

### Assembly of Discovery and Classification Subsets

**Fig. 1:**
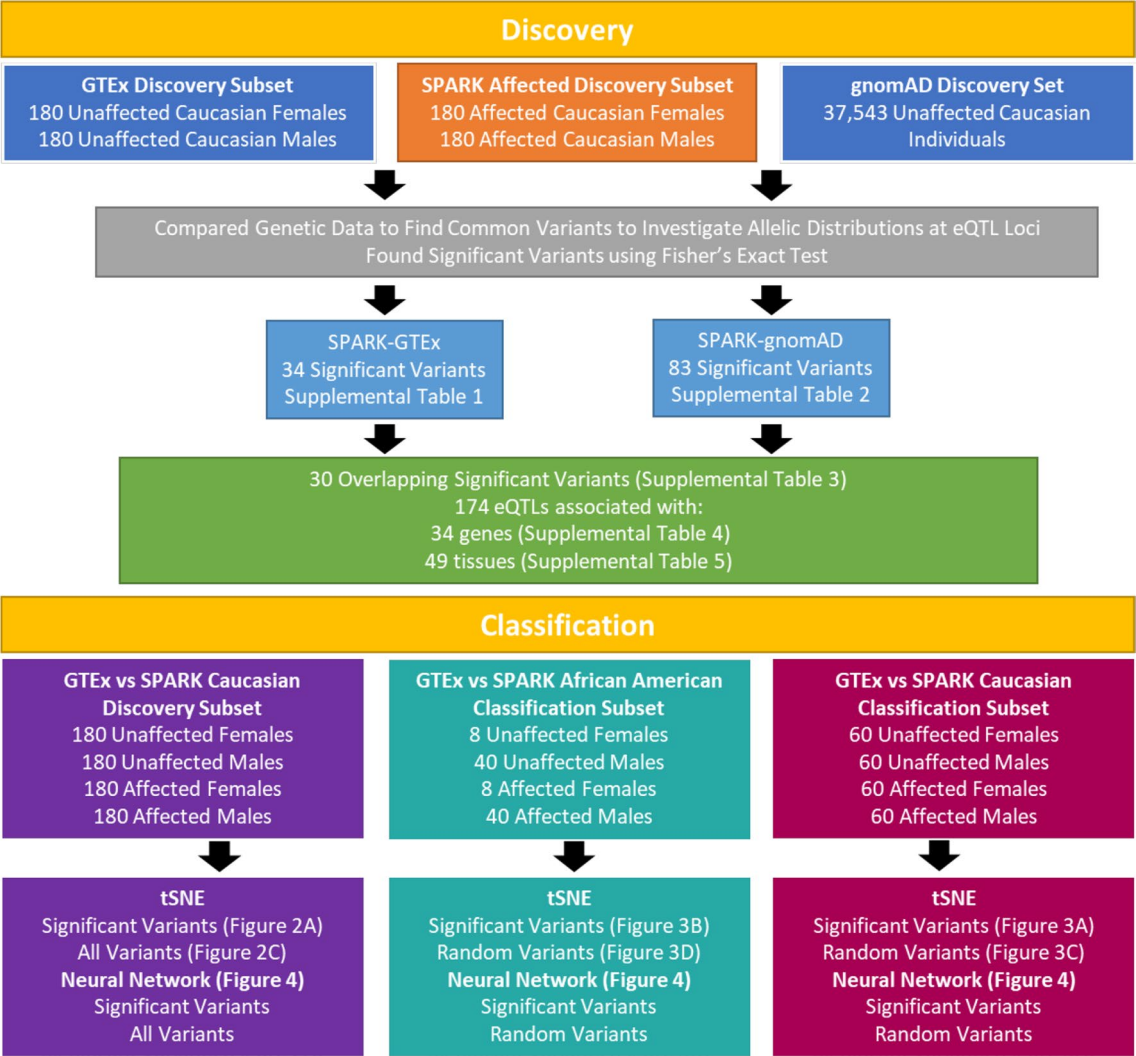
ASD Allele Discovery Workflow. This study was broken into two stages: Discovery and Classification. In the Discovery stage, two experiments were conducted to investigate variants with significantly different allele distributions between ASD-affected and ASD-unaffected individuals at eQTL loci: a SPARK-GTEx experiment and a SPARK-gnomAD experiment. At each eQTL associated variant, a Fisher’s Exact Test was performed to obtain two separate lists of variants of interest (34 for the SPARK-GTEx experiment and 83 for the SPARK-gnomAD experiment). The variants found in both experiments make up the final list of 30 significant variants that were associated with 174 tissue-specific eQTLs. To investigate how well the 30 significant variants could classify ASD-affected and ASD-unaffected individuals, the Classification stage used tSNE and neural networks on existing and new subsets of individuals

The primary analysis of this study used ASD-affected individuals from SPARK and ASD-unaffected individuals from GTEx and gnomAD to identify variants with significantly different allele distributions at eQTL associated loci. An overview of the workflow can be seen in (Fig. 1). For the main Discovery stage experiments using gnomAD and GTEx control groups, the SPARK Caucasian Discovery subset was assembled by randomly picking 180 ASD-affected males and females from the group of 1647 described above. Similarly, 180 ASD-unaffected males and females were randomly selected from the GTEx Caucasian Discovery subset. As no individual-specific genetic information was available from gnomAD, all summary information for the NFE-non-neuro population was utilized for each variant. For the Classification stage experiments, two new subsets were introduced, the Caucasian and the African American Classification subsets. For the Caucasian Classification subset, 60 SPARK ASD-affected males and females and 60 GTEx ASD-unaffected males and females were randomly selected from those not included in the Discovery subsets, making up a total subset of 240 individuals. For the African American Classification subset, all 40 males and 8 females were selected from the African American SPARK dataset, and 40 males and 8 females were randomly chosen from the African American GTEx dataset, totaling 96 individuals. A severity subset was also created from the SPARK Caucasian Discovery matrix (Additional File 1: Supplemental Fig. 1). The severity subset included individuals from the SPARK Caucasian Discovery subset that had provided corresponding phenotypic information. Of the 360 ASD-affected individuals in the SPARK Caucasian Discovery subset, 271 completed all three phenotypic surveys (Developmental Coordination Disorder Questionnaire, Repetitive Behavior Scale-Revised, and Social Communication Questionnaire). The scores across all three surveys were added up and the 90 individuals with the highest and lowest scores (denoted as most and least severe, 180 individuals total) were used in conjunction with 90 random GTEx individuals from the original SPARK-GTEx Caucasian Discovery subset.

### Discovery Stage: Finding Significant Variants, eQTLs, and Genes

In the SPARK-GTEx and SPARK-gnomAD experiments within the Discovery stage, a loci summary report was constructed for each experiment that included the number of reference and alternate alleles for each variant across the ASD-affected and ASD-unaffected individuals. A Fisher’s Exact Test (*SciPy stats* module) was then performed on the corresponding allelic data for each variant, therefore assigning each one a p-value. The variants were sorted from lowest to highest based on their p-value and consequently ranked. To prevent false-positives, a Benjamini–Hochberg correction was applied at a false discovery rate of 1%. To do so, a critical value was calculated for each variant. Moving down the ranked list, the last variant in which the p-value was less than the critical value was noted, and its corresponding p-value was deemed to be the cutoff. All variants with a p-value less than the cutoff were then deemed *significant*. This yielded a list of variants for each experiment in which ASD-affected and ASD-unaffected individuals have significantly different allelic distributions (Additional File 2: Supplemental Table 1 and Supplemental Table 2). The variants found across both experiments were compared. The variants that met the significant qualifications in both the SPARK-GTEx experiment and the SPARK-gnomAD experiment created a list of consensus *significant variants* (Additional File 2: Supplemental Table 3). This analysis was additionally performed on the Discovery Stage male and female subgroups separately to potentially discover sex-specific eQTLs. However, no new variants were identified. The list of significant variants was cross-referenced with the GTEx eQTLs (with a qval < 0.05) to make a list of *significant eQTLs* (Additional File 2: Supplemental Table 4). The information for tissue-specific information for associated eQTLs is also available (Additional File 2: Supplemental Table 4). The genes associated with the significant eQTLs were extracted to form a list of *significant genes*. These significant genes were further investigated using ToppFun to search for related molecular functions, biological functions, human phenotypes, and other associations among them, but no associations were deemed significant (FDR B&H p-value < 1E-5). A summary of the eQTLs and variants analyzed can be found in [Table 1]. The genes and their tissue associations are included in (Table 2).

**Table 1:** eQTL analysis summary

	Total eQTLs	eQTLs: GTEx qval < 0.05	Brain eQTLs: GTEx qval < 0.05	Variants Tested	Significant Variants	Significant eQTLs	Significant eQTLs in Brain
SPARK-gnomAD	1,207,976	475,829	98,934	12,933	83	300	52
SPARK-GTEx				12,789	34	182	27
Consensus				12,789	30	174	27

**Table 2:** Significant genes and their tissue associations

Gene name	Tissue
ACADVL	Adipose Subcutaneous, Adipose Visceral Omentum, Artery Aorta, Artery Tibial, Colon Sigmoid, Esophagus Gastroesophageal Junction, Nerve Tibial, Testis, Whole Blood
ANO5	Artery Aorta, Artery Tibial, Lung, Ovary
ASIC1	Heart Left Ventricle
BET1L	Artery Tibial, Colon Sigmoid, Colon Transverse, Pituitary, Skin Not Sun Exposed Suprapubic, Skin Sun Exposed Lower
CARNS1	Heart Left Ventricle
CES1	Adrenal Gland, Artery Aorta, Artery Tibial, Brain Amygdala, Brain Anterior cingulate cortex BA24, Brain Caudate basal ganglia, Brain Cerebellar Hemisphere, Brain Cerebellum, Brain Cortex, Brain Frontal Cortex BA9, Brain Hippocampus, Brain Hypothalamus, Brain Nucleus accumbens basal ganglia, Brain Putamen basal ganglia, Brain Spinal cord cervical c-1, Brain Substantia nigra, Cells Cultured fibroblasts, Colon Sigmoid, Colon Transverse, Esophagus Gastroesophageal Junction, Esophagus Mucosa, Esophagus Muscularis, Heart Atrial Appendage, Minor Salivary Gland, Nerve Tibial, Ovary, Pancreas, Pituitary, Prostate, Skin Not Sun Exposed Suprapubic, Skin Sun Exposed Lower leg, Spleen, Stomach, Testis
CES1P1	Adrenal Gland, Artery Aorta, Artery Coronary, Artery Tibial, Breast Mammary Tissue, Colon Sigmoid, Colon Transverse, Esophagus Gastroesophageal Junction, Esophagus Mucosa, Esophagus Muscularis, Heart Atrial Appendage, Lung, Minor Salivary Gland, Ovary, Prostate, Stomach, Testis, Thyroid, Uterus, Whole Blood
CIDEA	Esophagus Mucosa
CNTN2	Skin Sun Exposed Lower leg
CRELD2	Liver
GBA	Cells Cultured fibroblasts, Lung, Spleen, Thyroid
KLRC3	Lung, Spleen, Whole Blood
KLRC4	Adipose Subcutaneous, Esophagus Gastroesophageal Junction
KRTAP5-9	Lung
LCE4A	Skin Sun Exposed Lower leg
LINC02102	Nerve Tibial
MAN2C1	Adipose Subcutaneous, Adipose Visceral Omentum, Adrenal Gland, Artery Aorta, Artery Tibial, Brain Anterior cingulate cortex BA24, Brain Caudate basal ganglia, Brain Cortex, Brain Hippocampus, Brain Putamen basal ganglia, Brain Spinal cord cervical c-1, Cells Cultured fibroblasts, Cells EBV-transformed lymphocytes, Colon Sigmoid, Colon Transverse, Esophagus Gastroesophageal Junction, Esophagus Mucosa, Esophagus Muscularis, Heart Left Ventricle, Kidney Cortex, Lung, Minor Salivary Gland, Muscle Skeletal, Nerve Tibial, Pancreas, Pituitary, Prostate, Skin Not Sun Exposed Suprapubic, Skin Sun Exposed Lower leg, Small Intestine Terminal Ileum, Spleen, Stomach, Testis, Thyroid, Vagina, Whole Blood
NQO2	Kidney Cortex
PAPOLB	Testis
PMPCB	Thyroid
POLR1B	Brain Putamen basal ganglia, Cells EBV-transformed lymphocytes
POM121B	Esophagus Mucosa
PTGIS	Nerve Tibial
RP11-817O13.8	Adipose Subcutaneous, Breast Mammary Tissue, Esophagus Gastroesophageal Junction
SIRPB1	Brain Anterior cingulate cortex BA24, Brain Caudate basal ganglia, Brain Cortex, Brain Nucleus accumbens basal ganglia, Breast Mammary Tissue, Cells Cultured fibroblasts, Cells EBV-transformed lymphocytes, Heart Atrial Appendage, Pituitary
SLC5A10	Testis
SNORD3B-2	Adipose Subcutaneous
SPATA31C1	Brain Cortex, Pituitary
SYT5	Brain Caudate basal ganglia, Brain Putamen basal ganglia
TTL	Esophagus Gastroesophageal Junction, Skin Not Sun Exposed Suprapubic
VARS2	Cells Cultured fibroblasts, Cells EBV-transformed lymphocytes, Colon Transverse, Heart Left Ventricle, Skin Sun Exposed Lower leg, Spleen, Whole Blood
XXbac-BPG181B23.7	Vagina
ZNF490	Adipose Subcutaneous, Artery Aorta, Artery Tibial, Breast Mammary Tissue, Esophagus Gastroesophageal Junction, Esophagus Muscularis, Nerve Tibial, Whole Blood
ZNF587B	Colon Sigmoid

### Expression of Significant Genes in Brain Tissues

**Fig. 2:**
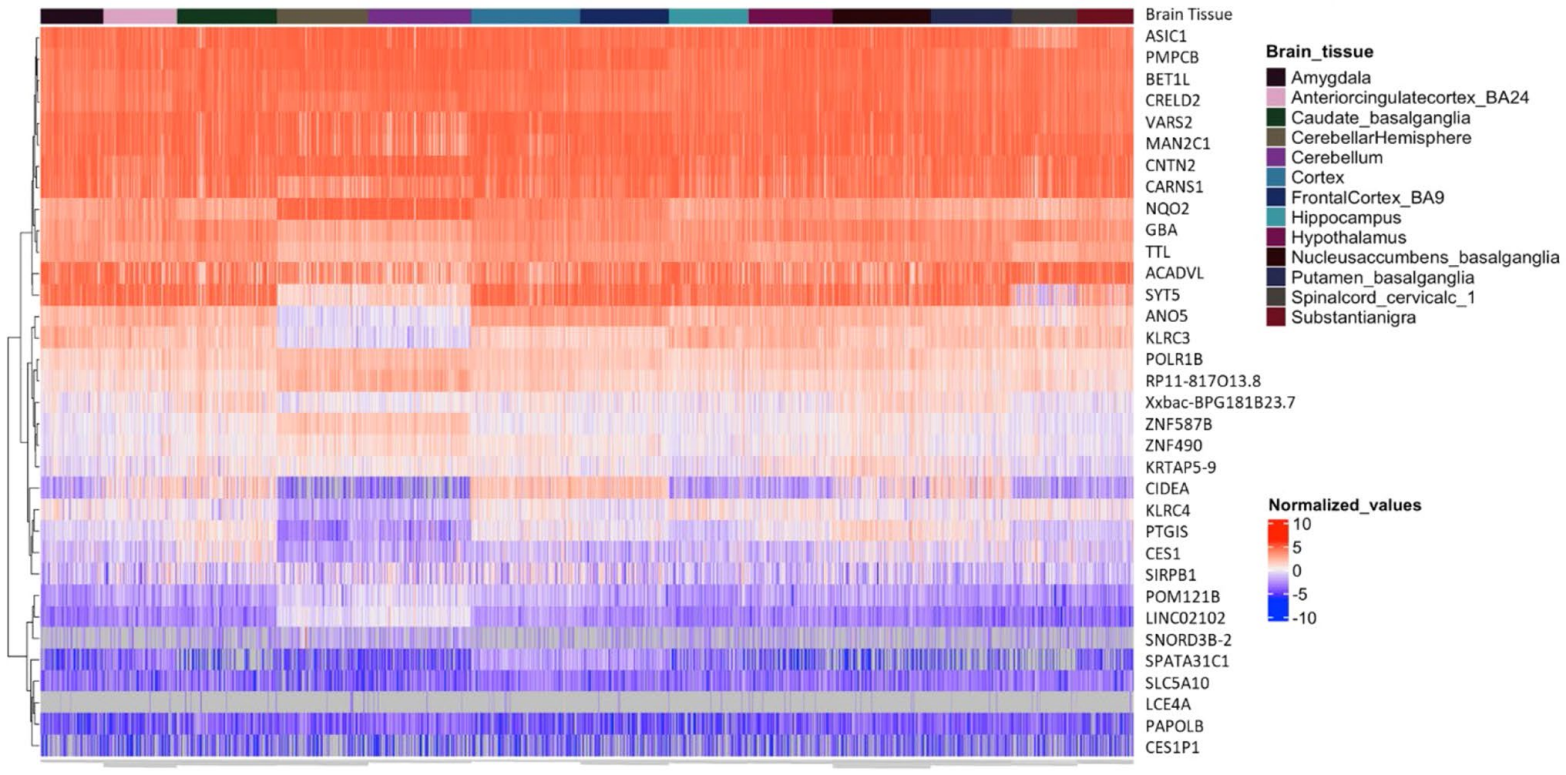
Heatmap Depicting Gene Expression of Significant Genes in Brain Tissues. Expression values of the significant genes were derived from brain samples of the GTEx dataset, arranged into a matrix, and subsequently normalized. The darkly colored blocks at the top of the figure represent the brain region from which the sample was taken and genes were sorted by hierarchical clustering. In the heatmap red represent higher levels of expression, while blue represents lower levels of expression. For example, ANO5 and KLRC3 had lower levels of expression in the cerebellum and cerebellar hemisphere compared to other tissues

To investigate how the significant genes were expressed in the brain, a matrix of TPM expression values for 2,564 GTEx brain samples from 13 brain tissues was downloaded from the GTEx portal and processed [https://gtexportal.org/home/datasets]. The RNAseq expression values were log2 transformed, quantile normalized, and 78 outliers were removed (Kolmogorov–Smirnov test (KS Dval > 0.15) using GEMprep [https://github.com/SystemsGenetics/GEMprep.git]. This matrix was used to make a heatmap (generated using the R (v4.3) package ComplexHeatmap) (Fig. 2). Genes (rows) were grouped using hierarchical clustering. Columns were ordered based on the 13 brain tissues as indicated on the heatmap.

### Variant Associations with Active Brain Regulatory Regions

We were interested in finding more relevant associations for the significant eQTLs, especially related to the brain. This was done by downloading files of interest from PsychENCODE [http://resource.psychencode.org]. The enhancers are active specifically in the prefrontal cortex (PFC), while the three acetylation peak files represent the PFC, the temporal cortex (TC), and the cerebellar cortex (CBC). The PFC enhancers were available in hg38, but the acetylation peaks were published in hg19. Therefore, the acetylation peak coordinates were transferred to hg38 using UCSC’s liftOver [https://genome.ucsc.edu/cgi-bin/hgLiftOver]. After all features were in the same genome build, the variants and genes of the significant eQTLs were compared to the brain-specific associations. The associations were noted in (Table 3) and Supplementary Table 4.

**Table 3:** Significant genes with at least 1 brain association

Gene name	Brain/endocrine association
ACADVL	Associated Variant within CBC Acetylation Peak
ANO5	Associated Variant within PFC Acetylation Peak and TC Acetylation Peak
BET1L	eQTL Association with Pituitary Gland
CARNS1	PsychENCODE ASD DEG; lower in ASD
CES1	eQTL Associations with 13 Brain Tissues, Adrenal Gland, and Pituitary Gland
CES1P1	eQTL Association with Adrenal Gland
CIDEA	PsychENCODE ASD DEG; lower in ASD
KLRC3	Associated Variant in PFC Acetylation Peak and TC Acetylation Peak
KLRC4	Associated Variant in PFC Acetylation Peak and TC Acetylation Peak
MAN2C1	eQTL Associations with Adrenal Gland, 6 Brain Tissues, and Pituitary Gland
POLR1B	Associated Variant within PFC Acetylation Peak, TC Acetylation Peak, and CBC Acetylation Park; eQTL Association with Brain Putamen Basal Ganglia
SIRPB1	Associated Variant within PsychENCODE Enhancer and Gene/Variant Assocation with PsychENCODE eQTL; eQTL Association with 4 Brain Tissues and Pituitary Gland
SPATA31C1	eQTL Associations with Brain Cortex and Pituitary Gland
SYT5	eQTL Associations with Brain Putamen and Caudate Basal Ganglia

### Classification Stage: tSNE

**Fig. 3:**
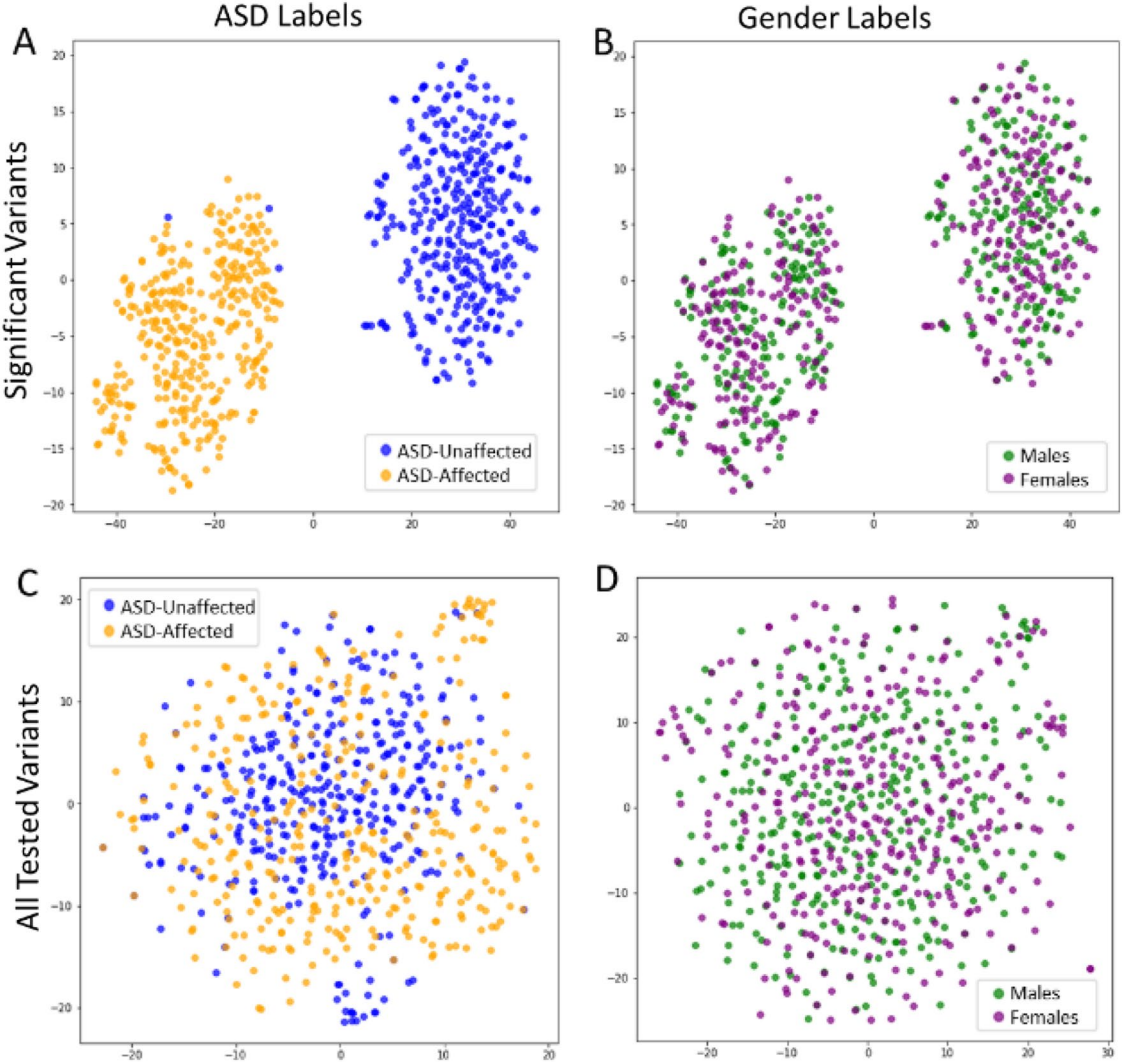
tSNE of the Discovery Subset at All Tested eQTL Variants and Significant Variants. tSNE allows for the visualization and distribution of individuals in the Caucasian Discovery subset based on their genotype profiles at the significant variants found in the Discovery stage of this study (N = 30, panels A and B) and all variants tested (N = 12,789, panels C and D). In these tSNE plots, each dot represents an individual. In plots A and C the individuals were colored based on their ASD status, while in plots B and D, individuals were colored based on sex

**Fig. 4:**
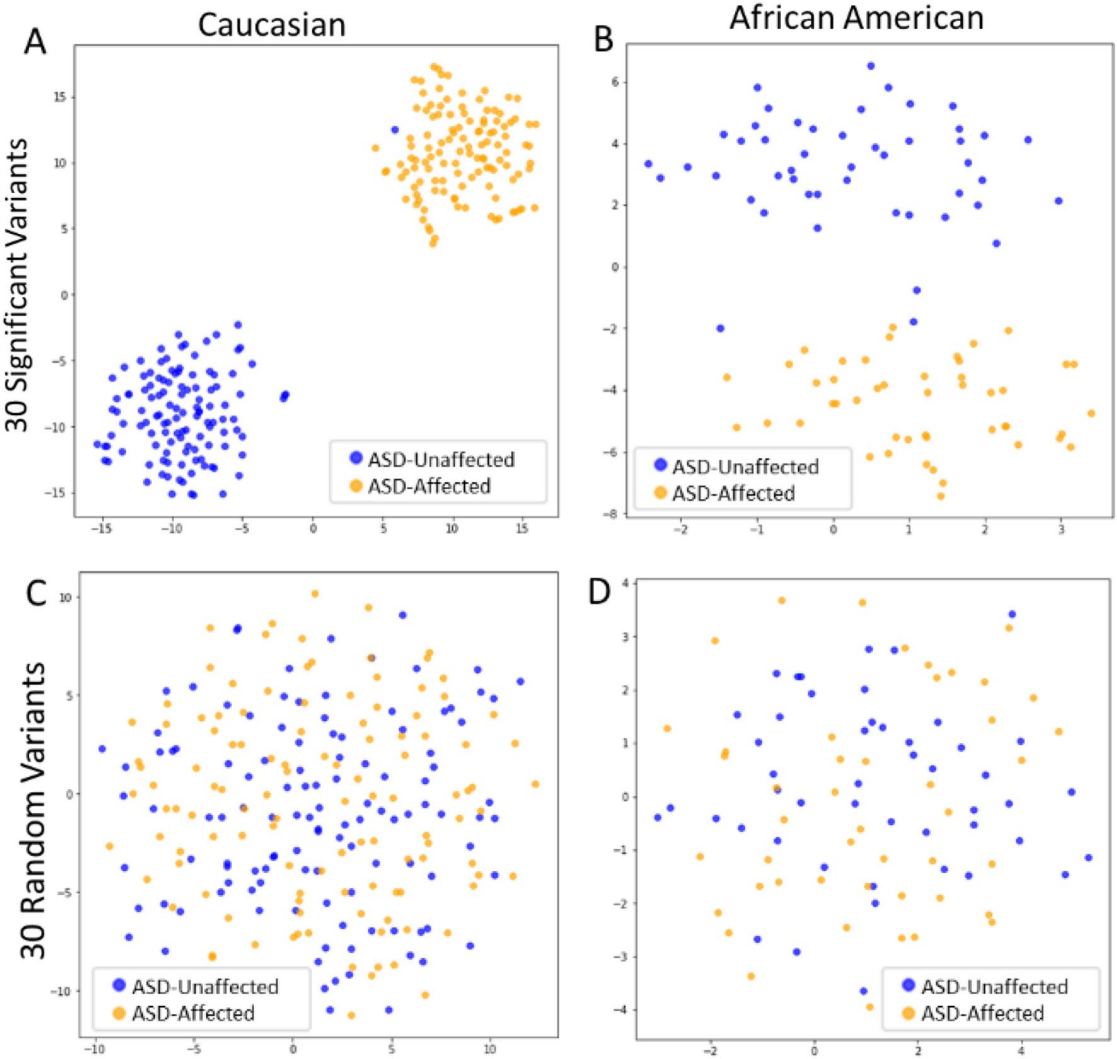
tSNE of Caucasian and African American Classification Subsets at Significant and Random Variants. Using tSNE the distribution of Caucasian and African American Classification subsets can be seen when using the genotype profiles of the 30 significant variants as input (panels A and B), compared to using that of 30 random variants (panels C and D). Each dot represents an individual, and the color of each dot represents that individual’s ASD status

The SPARK-GTEx Caucasian Discovery subset, SPARK-GTEx Caucasian Classification subset, and SPARK-GTEx African American Classification subset all underwent visualization using tSNE. For the SPARK-GTEx Caucasian Discovery subset of individuals, the genotype profiles of the significant variants and all variants tested were used as inputs (Fig. 3). For the Classification subsets, instead of using all tested variants as a comparison, 30 variants were randomly chosen (Fig. 4). For each tSNE plot, the input matrix first underwent principal component analysis (PCA), which was then followed by tSNE. The *sklearn* Python package was used for both computational processes.

### Classification Stage: Neural Network

**Fig. 5:**
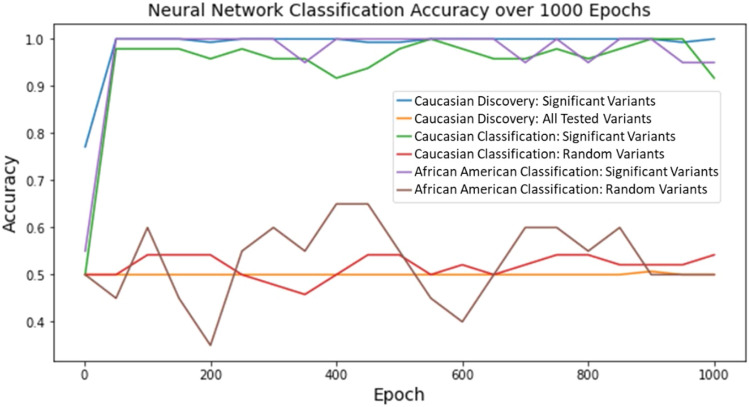
Neural Network Classification Accuracy for Various Datasets over 1,000 Epochs. A MLP neural network was used to observe how individuals could be classified into ASD-affected and ASD-unaffected groups using their genotype profiles across different data inputs. Within all three subsets of individuals (Caucasian Discovery subset, Caucasian Classification subset, and African American Classification subset) using the genotype profiles of the 30 significant variants classified individuals at a higher accuracy compared to random variants (N = 30) and all variants tested (N = 12,789)

Initially, the data and label file were loaded and divided into an 80–20 train-test split. The data was a two-dimensional matrix of genotypes that corresponded to each individual at the variants of interest. The genotypes were a discrete value ranging from −1 to 2. The label file mapped the individual to its label (“affected” or “unaffected”). The input values were then converted to one-hot encoding. Next, a multilayer perceptron model was constructed with *PyTorch* for binary classification. The model consisted of three hidden layers and an output layer with 120, 84, 10, and 1 neuron respectively. One dropout layer was placed between each hidden layer and every hidden layer applied the ReLU activation function. The model was trained on 80% of the input data using a batch size of 8 for 1,000 epochs. The BCEWithLogitsLoss function was used during training to combine a sigmoid layer and the loss function for binary classification. For every epoch, after the model updates its weights, a forward pass was done on the test set to calculate the accuracy. The sigmoid activation function was used to estimate the probability that the sample belongs to the “affected” or “unaffected” class. Once finished, the accuracy of the test set was plotted every 50 epochs (Fig. 5).

### Secondary Analysis: Finding Significant Variants Within SPARK Dataset

**Fig. 6:**
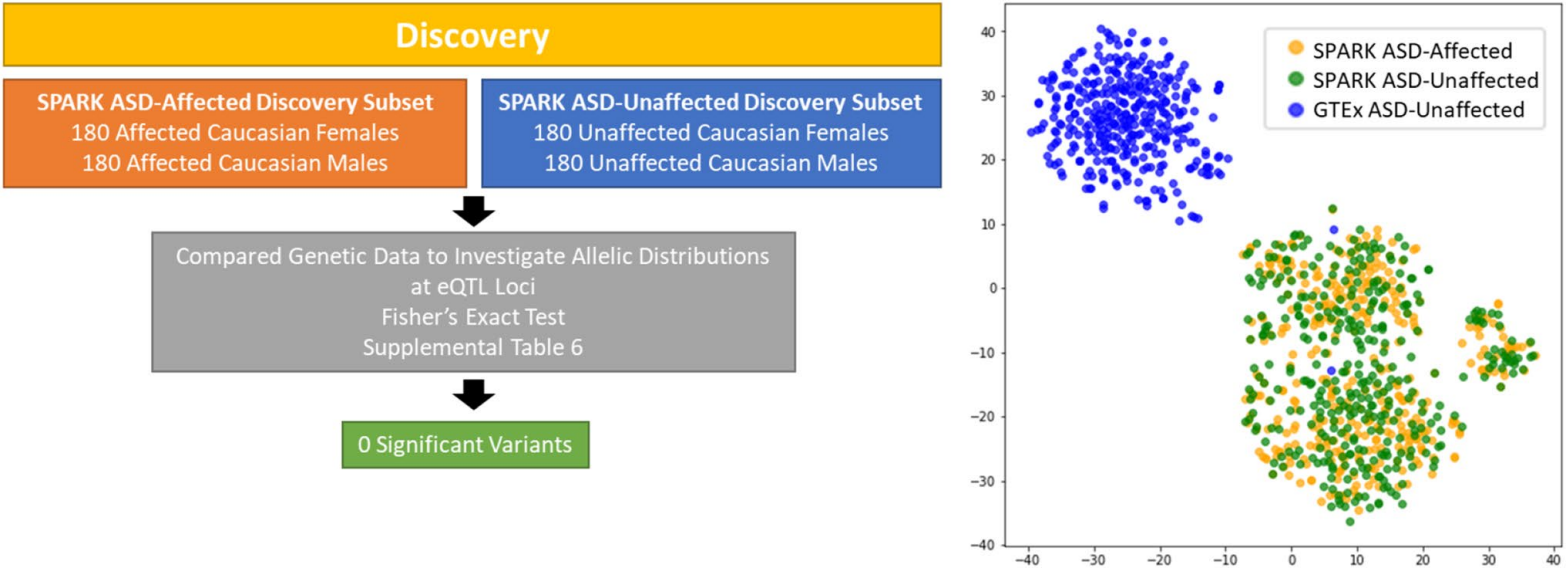
Analysis Investigating Allelic Distributions between SPARK ASD-Affected Individuals and Unrelated SPARK ASD-Unaffected Individuals. We investigated if the significant variants found in the initial Discovery stage of this analysis carried over when comparing SPARK ASD-affected individuals to unrelated ASD-unaffected individuals with ASD-affected siblings. When using a Fisher’s Exact Test to analyze the allelic distributions at eQTL variants, no variants had a significantly different distribution between the ASD-affected and ASD-unaffected subsets. A tSNE plot was then used to visualize the distribution of Caucasian SPARK ASD-affected individuals, Caucasian SPARK ASD-unaffected individuals, and GTEx ASD-unaffected individuals at the previously identified significant variants

To conclude the study a final analysis was performed comparing the allelic distributions of SPARK-ASD affected individuals and SPARK-ASD unaffected individuals at eQTL variants (Fig. 6). The same population of 360 Caucasian SPARK ASD-affected individuals used in the primary Discovery analysis was used again here. The group of SPARK ASD-unaffected individuals were siblings of individuals diagnosed with ASD that were not included in the Discovery subset. Therefore, they were not diagnosed with ASD, but they have an ASD-affected sibling who was not in the Discovery subset, so no familial substructure would exist between the two groups. A total of 1,651 ASD-unaffected siblings (855 males and 796 females) met these criteria. Of those 1,651 individuals, 180 males and 180 females were randomly chosen. The same analysis used in the primary Discovery analysis was completed again here. A Fisher’s Exact Test was completed at each eQTL variant to compare the distribution of alleles between the ASD-affected and ASD-unaffected groups, a p-value was assigned, and a Benjamini–Hochberg procedure was used to correct for multiple hypothesis testing. After correction, no variants were identified in which there was a significant difference in the allele distribution across the two groups (Additional File 2: Supplemental Table 6). To visualize this, a tSNE plot was used with the input of genotype profiles at previously discovered significant variants for the SPARK Caucasian ASD-affected individuals, SPARK Caucasian ASD-unaffected individuals, and GTEx Caucasian ASD-unaffected individuals (Fig. 6). The PCA and tSNE processes were both from the *sklearn* Python package.

## Results

In this study we were interested in finding alleles associated with ASD at eQTL loci. An overview of the workflow can be seen in Fig. 1. All tissue-specific eQTLs were downloaded from GTEx (N = 1,207,976 eQTLs), however only those that met the significance threshold recommended by GTEx (qval < 0.05) were retained for further analysis (Table 1). Of the 475,829 eQTLs that met the significance threshold, 98,934 were associated with a brain tissue (Table 1). The variants associated with the 475,829 eQTLs were extracted and used for the following analysis.

The primary analysis was organized into a Discovery stage and a Classification stage. In the Discovery stage, variants with significantly different allelic distributions between ASD-affected and ASD-unaffected individuals were discovered. In the Classification stage, we used tSNE and a neural network to observe the classification of ASD-affected and ASD-unaffected individuals into their respective groups based on the genotype profiles at the significant variants. The Discovery stage was composed of two experiments: investigating the SPARK and GTEx datasets as well as the SPARK and gnomAD datasets. The SPARK dataset was made up of 360 ASD-affected individuals. For the controls, one experiment used 360 GTEx ASD-unaffected individuals and the other used 37,543 gnomAD ASD-unaffected individuals. From the SPARK and GTEx datasets the 360 individuals were chosen at random, and only one member of each family was chosen to avoid familial substructures. In the gnomAD dataset, no individual-specific data was available, so the population-specific genetic data for each variant was used.

Once the experimental and control groups were determined, the variants from the GTEx eQTLs were compared to the genomic information available in each control dataset. For the SPARK and GTEx datasets, variants had to have genotype calls for at least 90% of the Discovery individuals to be considered for further analysis. The SPARK-gnomAD experiment was able to investigate 12,933 eQTL-associated variants, while the SPARK-GTEx experiment investigated 12,789 eQTL-associated variants (Table 1).

For each experiment, every variant underwent a Fisher’s Exact Test to compare the number of reference and alternate alleles between the ASD-affected and ASD-unaffected individuals. The variants were then ranked by their p-values from lowest to highest. As each variant-ASD association test represents a single hypothesis, we corrected for false discovery using the Benjamini–Hochberg procedure and a false discovery rate of 1%. For each variant, the critical value was calculated (critical value = (rank/total number of variants) * 0.01). We then moved down the ranked variant list to find the last variant where the p-value was less than the critical value. The variant information (including the reference allele counts, alternate allele counts, ranks, p-values and critical values) for the SPARK-GTEx experiment and SPARK-gnomAD experiment is also available (Additional File 2: Supplemental Table 1 and Supplemental Table 2).

The 34 significant variants discovered in the SPARK-GTEx experiment were then compared to the 83 significant variants from the SPARK-gnomAD experiment. There were 30 variants that were found to be significant among both experiments (Additional File 2: Supplemental Table 3). These were deemed to be the *significant variants*. These significant variants were then compared to the 475,829 GTEx eQTLs (with a q-val < 0.05) to find their gene and tissue associations. The significant variants aligned with a total of 174 tissue-specific eQTLs (Table 1, Additional File 2: Supplemental Table 4), representing 34 genes and 49 tissues. The 174 associated eQTLs were then known as *significant eQTLs*, and the 34 associated genes as *significant genes*. The significant genes and their tissue associations can be seen in Table 2. The expression levels of the significant genes across multiple brain tissues can be seen in Fig. 2. An eQTL summary by tissue can be seen in Additional File 2: Supplemental Table 5.

We were interested in any known brain associations among the significant eQTLs. PsychENCODE has published a list of active brain enhancers, lists of acetylation peaks specific to the pre-frontal cortex (PFC), cerebellar cortex (CBC), and temporal cortex (TC) areas of the brain, a list of active brain eQTLs, and a list of genes differentially expressed in ASD (Wang et al., 2018). These associations were downloaded and compared to the list of significant variants and significant genes. Fourteen of the significant genes had an association of interest. They were associated with a variant that fell within a brain regulatory region or were associated with a brain or endocrine tissue through GTEx. These genes and their relevant associations can be seen in Table 3.

To further investigate the classification accuracy of the genotype profiles of the significant variants, we used tSNE and a MLP neural network in the Classification stage of the study. The Classification stage consisted of three subsets of individuals: the Caucasian subset used in the Discovery stage, a new African American subset, and a new Caucasian subset. For this stage all the ASD-affected individuals came from the SPARK dataset while all the ASD-unaffected individuals came from the GTEx dataset. The Caucasian Discovery subset was made up of the same individuals used earlier (180 ASD-unaffected females, 180 ASD-unaffected males, 180 ASD-affected females, 180 ASD-affected males), while the African American Classification subset (8 ASD-unaffected females, 40 ASD-unaffected males, 8 ASD-affected females, 40 ASD-affected males) and the Caucasian Classification subset (60 ASD-unaffected females, 60 ASD-unaffected males, 60 ASD-affected females, 60 ASD-affected males) introduced new individuals. Two new subsets were included to verify that the significant variants found in the Discovery stage were not solely an artifact of the random group of Caucasian individuals in the Discovery stage but could also apply to a separate group of Caucasians as well as an additional population.

First, tSNE was used to visualize how individuals from the Caucasian Discovery subset segregated based on their genotypic profiles at the significant variants and all variants tested. In Fig. 3A, the genotype profiles at the significant variants were able to segregate ASD-affected (blue dots) and ASD-unaffected individuals (orange dots) apart from a few GTEx outliers that clustered with the SPARK individuals. However, this was not true when considering the genotype profiles at all variants tested as seen in Fig. 3C. To ensure that the individuals were not segregating by sex, sex labels were used in Fig. 3B and D: males (green dots) and females (purple dots). In Fig. 3B, there are no distinguishable clusters based on sex, which showed that the two main clusters in Fig. 3A were not sex related. In addition to sex labels, labels based on ASD severity can be found in Additional File 1: Supplemental Fig. 1. We observed that when reducing the number of variants to only those found to be significant, tSNE was able to separate individuals based on their genotype profiles and therefore their ASD status.

The tSNE approach employed above was used similarly for the African American and Caucasian Classification subsets. In Fig. 4 the distribution of individuals based on the genotype profiles at the 30 significant variants and 30 random variants can be seen. For both populations the genotype profiles at the significant variants (Fig. 4A and B) were able to separate ASD-affected and ASD-unaffected individuals better than the same number of random variants (Fig. 4C and D). The separation of African American ASD-affected and ASD-unaffected individuals in Fig. 4B was not as distinct as the Caucasian individuals in Fig. 4A. This could possibly be attributed to the lesser number of African American individuals available within the SPARK dataset.

The matrices used to create the tSNE plots in Figs. 3 and 4 were also used with a MLP neural network to observe how well it could classify individuals into ASD-affected and ASD-unaffected groups based on their genotype profiles at relevant loci (Fig. 5). Across all three groups of individuals (Caucasian Discovery, Caucasian Classification, and African American Classification) the classification accuracy based on the genotype profiles of the 30 significant variants was higher (ranging from 90 to 100%) than that of 30 random variants or all variants tested (accuracy ranging from 30 to 70%). The random and complete variant lists also have more sporadic fluctuations in accuracy. Nonetheless, their average classification accuracy centered around 50%, which is expected for a random classification of individuals into two groups.

Finally, we tested if the significant variants found using GTEx and gnomAD as control datasets would also be discovered if we used ASD-unaffected siblings from the SPARK dataset as the control. This is referred to as the secondary analysis in this study, an overview of which can be seen in Fig. 6. The same Caucasian Discovery subset was used in conjunction with 360 Caucasian ASD-unaffected siblings from the SPARK dataset (180 ASD-unaffected males, 180 ASD- unaffected females). While these ASD-unaffected controls were siblings of ASD-affected probands, there were enough individuals within the dataset to ensure that there were no familial relations within or between the ASD-affected and ASD-unaffected groups. The same approach was taken for this experiment as the initial Discovery stage of the study. For each variant investigated, 90% of individuals from the SPARK ASD-affected and SPARK ASD-unaffected subsets had to have a genotype call. A Fisher’s Exact Test was used at each variant to compare the allelic distributions of the ASD-affected and ASD-unaffected groups. Between these two groups, no significant variants were discovered after Benjamini–Hochberg correction. A tSNE plot was used to compare the segregation of SPARK ASD-affected, SPARK ASD-unaffected, and GTEx ASD-unaffected individuals at the 30 significant variants found in the earlier experiments (Fig. 6). The plot shows all SPARK individuals clustering together, while the GTEx ASD-unaffected individuals segregate separately except for two individuals (seen as two blue dots among the yellow and green).

## Discussion

In this study, we set out to identify variants at eQTL-associated loci with significantly different allele distributions between ASD-affected and ASD-unaffected individuals. Using controls from non-ASD datasets, 30 significant variants associated with 34 genes were identified. Interestingly, when using unrelated ASD-unaffected siblings of ASD-affected individuals, no variants had significantly different allele distributions. When visualizing the distribution of ASD-affected and ASD-unaffected individuals from the Caucasian Discovery subset, Caucasian Classification subset, and African American Classification subset in Figs. 3 and 4, ASD-affected and ASD-unaffected individuals were seen to easily segregate based on their genotype profiles at the 30 significant variants identified in the Discovery stage. It is unlikely that the ASD-affected and ASD-unaffected groups are separating based on any possible dataset bias. In Fig. 3A, several GTEx ASD-unaffected individuals (blue dots) separated with the SPARK ASD-affected individuals (orange dots) when analyzing the genotype profiles from the significant variants. If there were ascertainment bias (Lachance & Tishkoff, 2013), we would expect to see separation of the datasets in Figs. 3C,  4C, and D as well when observing the distribution of individuals at all variants tested or 30 random variants. It is possible that the few GTEx ASD-unaffected individuals that separated with the ASD-affected SPARK individuals (seen in Figs. 3, 4, 6, and Additional File 1: Supplemental Fig. 1) have ASD-affected relatives. That information was not included in the phenotypic surveys for the GTEx dataset but could be examined in future studies.

Another interesting characteristic found in several of the tSNE plots was the formation of clusters within the ASD-affected and ASD-related groups at significant variants. In Figs. 3, 6, and Additional File 1: Supplemental Fig. 1 there are three sub-clusters when observing individuals from the SPARK dataset. We tested to see if this sub-cluster formation was due to gender (Fig. 3B) or to ASD severity (Additional File 1: Supplemental Fig. 1), however neither trait segregated with the clusters. These clusters were also not distinctly present in Fig. 3C (when observing all variants) or Fig. 4 (when using the Discovery subsets). Because they were not present when using genotype profiles at all variants, it is likely something specific about these significant 30 variants that results in the formation of the sub-clusters. They were not seen in Fig. 4, but it is possible this was because of the fewer number of individuals in the Classification subsets, as there were 120 Caucasian ASD-affected individuals and 48 African American ASD-affected individuals in the Classification subsets, compared to 360 individuals in the Discovery subset. This is reinforced by the slight cluster formation seen in Additional File 1: Supplemental Fig. 1, which contained 90 less severe ASD-affected individuals and 90 more severe ASD-affected individuals. Therefore, the sub-cluster formation among ASD-affected individuals at the significant variants seems to become more distinct as the number of individuals increases. This is typical of tSNE visualization, where the patterns become clearer as the number of individuals increases. The sub-cluster formation at the significant variants can also be seen when SPARK ASD-related individuals were added in. In Fig. 6, the ASD-related individuals are segregating into sub-clusters along with the ASD-affected individuals, while the GTEx ASD-unaffected individuals do not. We hope to further investigate any other possible associations with these sub-clusters in future studies.

The datasets that underwent tSNE visualization were also subject to a neural network analysis where a machine learning model predicted if an individual was ASD-affected or ASD-unaffected based on their genotype profile. The model was able to classify individuals with a high accuracy when inputting the genotypes for the significant variants for all three groups tested: Caucasian Discovery subset, Caucasian Classification subset, and African American Classification subset. When using the genotypes for all variants or random variants, the classification accuracy was much lower, fluctuating between 30 and 70%. For these results, the neural network predictions seem to become more stable as more individuals are added. For example, the Caucasian Discovery subset has the most individuals (720 total) and the most consistent trends in the classification accuracy, while the African American Classification subset has the least individuals (96 total) and the least consistency in classification accuracy when using genotypes at 30 random variants as input.

In the secondary analysis all variants were tested to find those that had significantly different allele distributions between SPARK ASD-affected and SPARK ASD-unaffected individuals, but no variants were identified as significant. To visualize this, a tSNE plot was used for the genotype profiles at the significant variants of the primary analysis for 360 Caucasian SPARK ASD-affected, 360 Caucasian SPARK ASD-unaffected, and 360 Caucasian GTEx ASD-unaffected individuals. In Fig. 6, the SPARK ASD-unaffected individuals can be seen segregating with the SPARK ASD-affected individuals into sub-clusters rather than with the GTEx ASD-unaffected individuals. The SPARK ASD-unaffected individuals were siblings of SPARK ASD-affected individuals that were not included in these analyses. However, if we can assume that the SPARK ASD-affected siblings of the SPARK ASD-unaffected group follow similar genotypic trends as those in the Discovery and Classification subsets, then it is fair to say that the ASD-unaffected siblings are more likely to follow the ASD genotype pattern because of the inherent familial relationships compared to unrelated controls. It is possible that the significant variants identified in this study contribute to the hypothesis that the accumulation of otherwise common variants can also confer ASD risk. Previous research has also shown that ASD-unaffected siblings of ASD-affected siblings can still show milder symptoms of ASD, even as high as 15% of siblings exhibiting a ‘broad autism phenotype’ (Bolton et al., 1994; Gamliel et al., 2007; Losh et al., 2009). Nonetheless, there seems to be no significant genetic differences between ASD-affected individuals and unrelated ASD-unaffected siblings at the variants we have identified.

The significant variants in this study were associated with 174 tissue-specific eQTLs derived from the GTEx database. Based on the log2aFC information of each variant and the allele distributions between ASD-affected and ASD-unaffected individuals, the direction of expression in ASD can be determined. Of the 174 eQTLs, 96 had higher gene expression in their associated tissue compared to 78 eQTLs with lower gene expression (Additional File 2: Supplemental Table 4). When looking solely at the 27 eQTLs associated with a brain tissue, 8 had lower gene expression in ASD individuals while 19 had higher gene expression (Table 1, Additional File 2: Supplemental Table 4). CES1 and MAN2C1 had the most GTEx brain associations. CES1, a drug metabolizer, has been shown to have significantly different mRNA levels in peripheral leukocytes of ASD-affected individuals and unrelated ASD-unaffected mothers of ASD-affected individuals, compared to controls (Johnson et al., 2013; Kuwano et al., 2011). Microdeletions in the area surrounding MAN2C1 have been linked to gene expression of MAN2C1 in the brain, a lower verbal and non-verbal IQ, and ASD (Cáceres et al., 2016; McInnes et al., 2010; Roetzer et al., 2010).

When comparing the significant eQTLs to data published by PsychENCODE, more brain related associations were found (Table 3). For example, POLR1B is associated with a variant that falls into an acetylation peak that is active in the PFC, TC, and CBC. POLR1B is a target of a microRNA (miR-365a-3p) that has been shown to be upregulated in ASD-affected individuals (Kichukova et al., 2016). The variant associated with SIRPB1 falls within a PsychENCODE enhancer and has been identified as an eQTL in the brain. SIRPB1 was deleted in a case study of monozygotic ASD twins, and has been linked with impulsive-disinhibited personality, meaning the individuals struggle to control their impulses, which has been implicated in ASD (Hill, 2004; Laplana et al., 2014a, 2014b; Laplana et al., 2014a, 2014b). One other example of an association with PsychENCODE can be found in the CIDEA gene, which was identified by our study and was shown to be significantly down-regulated in ASD-affected individuals by PsychENCODE. Additionally, CIDEA has shown to be down-regulated in obesity and associated with ADHD, both of which often appear in tandem with ASD (Cortese & Vincenzi, 2012; Croen et al., 2015; Kweon et al., 2018; Lee & Ousley, 2006; Nordström et al., 2005). In total, the eQTLs described here are evidence of tissue-specific regulation across both brain and non-brain tissues. While it is likely that neurological traits originate in the brain, ASD-affected individuals share common non-neurological traits as well. It is possible that eQTLs are one avenue in which a variant can result in changes of gene expression across multiple tissues, like can be observed for genes CES1 and MAN2C1 in this study. The significant variants and eQTLs discovered here will need to be further investigated to elucidate if a global regulatory variant and its consequent tissue-specific changes could be linked to ASD as a possible mechanism.

## Limitations

One limitation of this study was the scope of the genome we were able to investigate. Because the SPARK genotyping data was centered within and around genes, there are possibly more significant variants in intragenic regions that we were not able to investigate. An additional limitation is the population of ASD-affected individuals that we chose to study. By removing individuals with environmental exposures and those that had potentially inherited a large-scale genetic change from an ASD-affected parent, we likely lost statistical power and the opportunity to discover additional variants. We hope future studies will further investigate these regions and individuals.

## Conclusion

ASD is a common neurological disorder characterized by a variety of phenotypic traits and genetic associations. Here we investigated allelic distributions of variants associated with tissue-specific eQTLs between an ASD-affected population and two outside control populations to discover 30 significant variants. A neural network was then used to classify ASD-affected and ASD-unaffected individuals based on the genotypic profiles at the significant variants. For both the Caucasian and African American populations, the significant variants were able to correctly assign individuals to their respective ‘ASD-affected’ or ‘ASD-unaffected’ group with a higher accuracy (90–100%) compared to 30 random variants (30–70% accuracy). These significant variants were associated with 34 genes and 49 tissues for a total of 174 tissue-specific eQTLs. Comparison of these features with PsychENCODE, a comprehensive brain and ASD resource, showed that several of these variants exist within brain-specific regulatory regions and several genes had been previously identified as differentially expressed in ASD-affected individuals. Further research will be needed to validate if the eQTLs discovered here and the consequent changes in tissue-specific gene expression can result in the myriad of brain and non-brain phenotypic traits seen in ASD-affected individuals.

## Supplementary Information

Below is the link to the electronic supplementary material.

Supplementary file1 (PPTX 203 kb)

Supplementary file2 (XLSX 3900 kb)

## Data Availability

All data generated or analyzed during this study are included in this published article and its additional files. The eQTLs from RNA expression data from GTEx are available at [https://gtexportal.org/home/datasets]. The gnomAD data are publicly available for download at [https://gnomad.broadinstitute.org/downloads]. PsychENCODE data used in this study are publicly available for download at [http://resource.psychencode.org]. The individual-specific SPARK and GTEx genotype data that support the findings of this study are available but restrictions apply to the availability of these data, which were used under license for the current study, and so are not publicly available.
