# Supplementary material for: Discovery of eQTL Alleles Associated with Autism Spectrum Disorder: A Case–Control Study
Source: J Autism Dev Disord. 2022 Jun 23;53(9):3595–612. doi: 10.1007/s10803-022-05631-x (PMC10465380; doi:10.1007/s10803-022-05631-x)
Supplement: Supplementary file 1 — Supplementary file1 (PPTX 203 kb) [file 10803_2022_5631_MOESM1_ESM.pptx]

## Slide 1
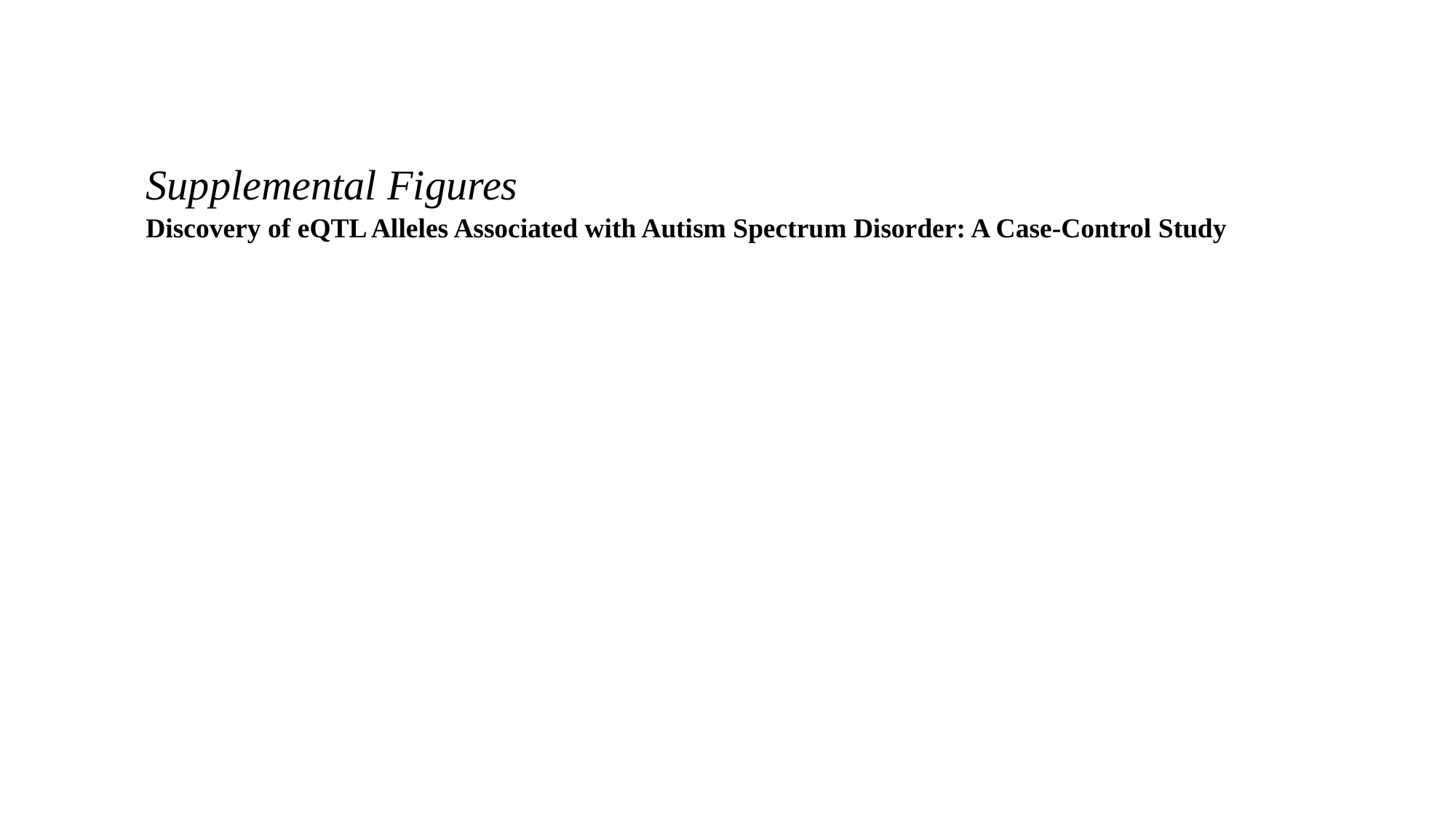

Supplemental Figures
Discovery of eQTL Alleles Associated with Autism Spectrum Disorder: A Case-Control Study

## Slide 2
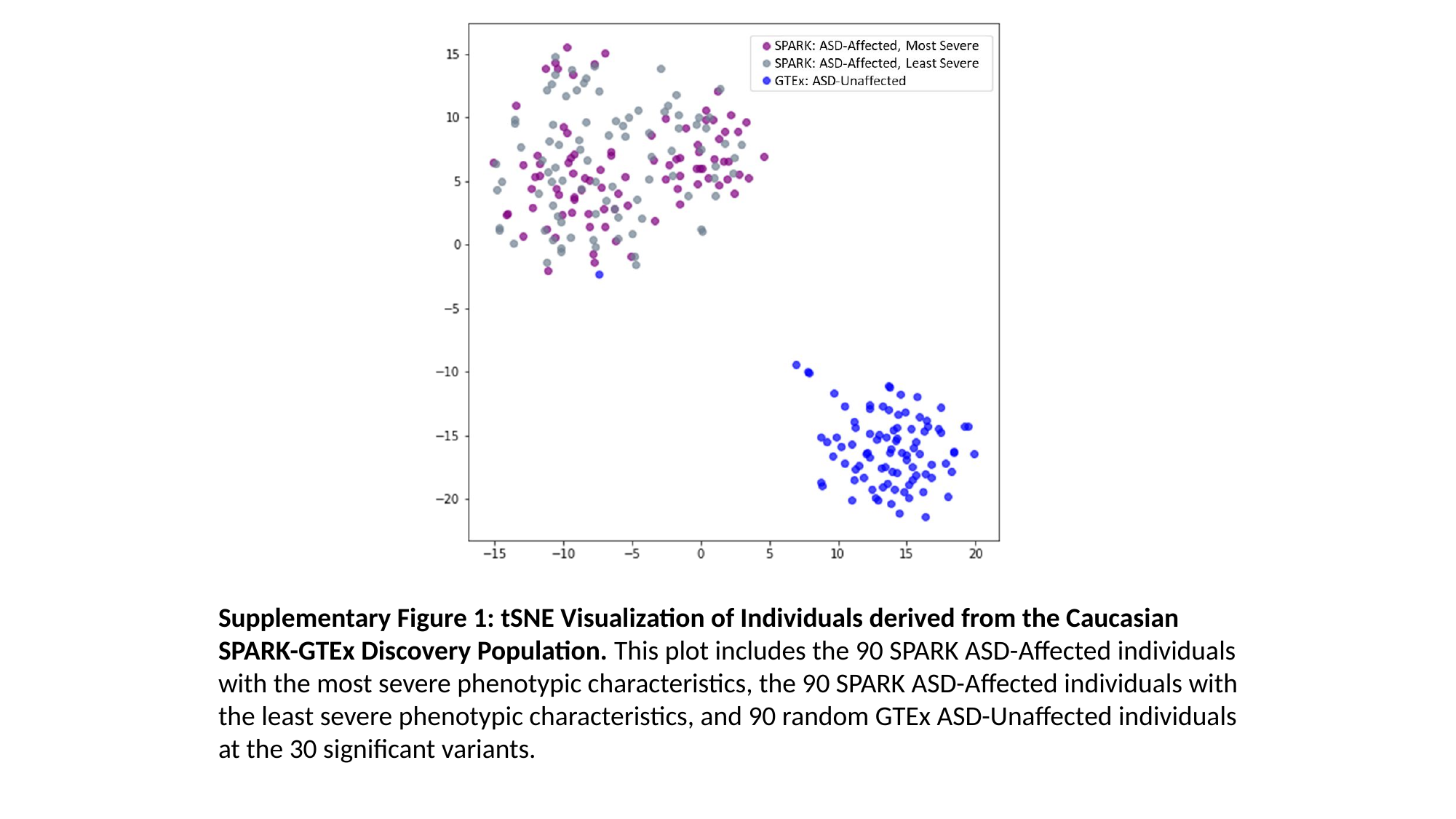

Supplementary Figure 1: tSNE Visualization of Individuals derived from the Caucasian SPARK-GTEx Discovery Population. This plot includes the 90 SPARK ASD-Affected individuals with the most severe phenotypic characteristics, the 90 SPARK ASD-Affected individuals with the least severe phenotypic characteristics, and 90 random GTEx ASD-Unaffected individuals at the 30 significant variants.
